# Neoantigen-Encoded Oncolytic Viruses as Personalized Cancer Vaccines

**DOI:** 10.3390/ph19030364

**Published:** 2026-02-26

**Authors:** Almohanad A. Alkayyal

**Affiliations:** Department of Medical Laboratory Technology, Faculty of Applied Medical Sciences, University of Tabuk, Tabuk 47512, Saudi Arabia; aalkayyal@ut.edu.sa

**Keywords:** oncolytic virus, neoantigen vaccine, personalized immunotherapy, in situ vaccination, immunogenic cell death, checkpoint blockade, HSV-1, vaccinia, adenovirus, Maraba, VSV, antigen presentation

## Abstract

Neoantigen vaccines have revitalized cancer vaccination by targeting tumor-specific mutant epitopes largely absent from central tolerance. Yet, clinical benefits remain inconsistent, in part because conventional vaccine platforms often do not reliably deliver antigens within an inflammatory tumor context, struggle to overcome immunosuppressive tumor microenvironments, and may not rapidly adapt to tumor heterogeneity and evolution. Oncolytic viruses (OVs) provide a mechanistically distinct route to “vaccinate in situ” by coupling tumor-selective infection and immunogenic cancer cell death with local innate immune activation, antigen release, and remodeling of the tumor microenvironment. In parallel, advances in sequencing, neoantigen prediction (e.g., updated NetMHCpan and MHCflurry tools as of 2025), and antigen presentation validation have enabled rational selection of patient-specific targets. At the same time, modern OV engineering supports insertion of neoantigen payloads and immune-modulatory transgenes. Here, we summarized principles that underpin neoantigen-encoded OVs as personalized cancer vaccines, emphasizing how OV adjuvanticity and antigenicity interact to drive priming, epitope spreading, and durable systemic immunity. We discussed major OV platforms with respect to payload capacity, expression control, manufacturability, and clinical track records, including lessons learned from approved or late-stage OVs such as talimogene laherparepvec (T-VEC) and teserpaturev/G47Δ. We also discussed design choices for encoding neoantigens (polyepitope strings, minigenes, long peptides; class I/II balancing), prioritizing translational biomarkers and immune-monitoring strategies, and outlining regulatory and GMP considerations for “platform-plus-variable insert” products. Finally, we propose a pragmatic clinical workflow for rapid personalization to maximize therapeutic index. Tightly integrating neoantigen science with immunovirotherapy, including recent 2025 preclinical advances like oncolytic adenovirus neoantigen delivery sensitizing low-TMB tumors to PD-1 blockade, could enable next-generation personalized cancer vaccines capable of converting “cold” tumors into responsive, systemically controlled disease.

## 1. Introduction

The resurgence of cancer vaccines has been driven by two converging advances: deep tumor profiling that enables patient-specific neoantigen identification and immunotherapy frameworks that reveal how T cells can mediate durable control when appropriately primed and sustained. Neoantigens in the form of peptides derived from tumor-specific somatic alterations are attractive vaccine targets because they are absent from the normal genome and thus are less constrained by central tolerance, increasing the probability of high-avidity T cell recognition [1,2]. Clinical studies in melanoma demonstrated that personalized neoantigen vaccines can induce polyfunctional CD4^+^ and CD8^+^ T cell responses, with evidence of tumor trafficking and long-term immune persistence [3,4,5]. Subsequent trials in multiple tumor types, including glioblastoma and non-small cell lung cancer, further established the feasibility of individualized neoantigen targeting, while highlighting real-world barriers such as immunosuppression, steroid exposure, and variable tumor antigen presentation [6,7]. Recent 3-year updates from KEYNOTE-942 confirm sustained recurrence-free and distant metastasis-free survival benefits with individualized mRNA neoantigen therapy plus pembrolizumab in resected high-risk melanoma.

In parallel, oncolytic virotherapy has matured from a niche concept into a clinically deployed modality. Talimogene laherparepvec (T-VEC), an engineered HSV-1 expressing GM-CSF, improved durable response rate versus GM-CSF in advanced melanoma (OPTiM phase III), validating that intratumoral viral therapy can translate into meaningful clinical benefit and systemic immune effects [8]. Other OV platforms such as vaccinia (JX594/pexa-vec), adenovirus derivatives, and reovirus have generated substantial translational evidence for tumor-selective replication, immune activation, and combinability with standard therapies [9,10,11]. Moreover, the conditional approval of G47Δ (teserpaturev) in Japan for malignant glioma underscored that genetically engineered herpesviruses can be clinically impactful even in immunologically challenging intracranial settings [12,13]. Recent Phase Ib trials have expanded T-VEC combinations, showing safety and efficacy with chemotherapy or endocrine therapy in breast cancer.

Despite this progress, each approach has limitations when deployed alone. Neoantigen vaccines can generate strong peripheral/circulating responses. Yet, these readouts do not always translate into effective tumor infiltration or clinical benefit, in part because T-cell exclusion and other microenvironmental barriers can prevent vaccine-primed T cells from reaching cancer cells [14,15,16]. Even when priming occurs, antitumor activity may be blunted by tumor antigen-presentation defects (e.g., MHC-I/APM downregulation), suppressive myeloid populations (TAMs/MDSCs), and checkpoint-associated dysfunctional/exhausted T-cell states [17,18,19,20,21,22,23,24]. Conversely, oncolytic viruses can inflame tumors and recruit immunity. Still, they do not necessarily enforce a consistent patient-specific antigen focus (often relying on endogenous antigen release and broad in situ vaccination effects). Their efficacy can be constrained by pre-existing or therapy-induced antiviral immunity that limits viral spread and/or repeat dosing [25,26]. Therefore, neoantigen-encoded OVs are conceptually compelling because they integrate individualized antigen programming directly into a tumor-localized inflammatory delivery vehicle, aiming to convert infected lesions into personalized vaccination sites that generate systemic, mutation-focused immunity and broaden epitope spreading [1,27].

## 2. Biological Rationale of Encoding Neoantigens into Oncolytic Viruses

A neoantigen-encoded OV can be viewed as a programmable immunovirotherapy platform with three synchronized mechanisms. First, direct viral infection and replication preferentially lyse tumor cells, releasing tumor-associated antigens and danger signals while reshaping local cytokine and chemokine landscapes. Second, viral innate sensing activates antigen-presenting cells (APCs), particularly dendritic cells (DCs), improving cross-presentation and T cell priming. Third, the engineered neoantigen payload ensures that priming is not left solely to stochastic antigen release and presentation, but instead includes defined mutation-specific epitopes that can be prioritized for clonality, expression, and predicted presentation. This “programmed plus in situ” logic aims to amplify both breadth and precision: encoded neoantigens bias the response toward clinically relevant mutant targets, while oncolysis supports antigen spreading to additional endogenous neoepitopes, potentially mitigating single-target escape [1,3,7].

The need to integrate CD4^+^ and CD8^+^ immunity is particularly important for OV payload design. In melanoma neoantigen vaccination, vaccine-induced CD4^+^ responses were frequently robust and polyfunctional, and coordinated helper responses are thought to support DC licensing, CD8^+^ memory formation, and the maintenance of durable effector function [3,4]. In the OV setting, CD4^+^ T-cell help becomes a force multiplier for neoantigen-specific CD8^+^ immunity because OV therapy creates a vaccination environment that is highly inflammatory, has repeated dosing, and antigen competition from viral epitopes. These factors can amplify immunodominance, skewing the response toward ’easy-to-see’ viral antigens rather than tumor-relevant neoantigens—unless CD4+ help is actively engineered into the system [28,29,30]. Mechanistically, the most established route is dendritic-cell (DC) licensing. When neoantigen-specific CD4^+^ T cells recognize their cognate peptide–MHC II on DCs, they deliver CD40L-mediated signals (plus cytokines) that upregulate DC costimulation and antigen-presentation competence, improving the quality of CD8^+^ priming (signal 2) and shaping differentiation away from short-lived, terminal effector states toward durable, recall-capable memory. In practical terms, licensed DCs drive better CD8^+^ expansion, enhance survival programming, and improve secondary responsiveness features we need in a platform where OV dosing and evolving tumor antigen landscapes repeatedly challenge T-cell fitness [28,31]. In addition, OV-driven innate cytokines (type I IFNs, inflammatory IL-12-family cues) can be a double-edged sword, supporting cross-priming but also pushing overly inflammatory differentiation. CD4 help acts as a stabilizer, sustaining effector function with IL-2 and reinforcing productive priming through CD40–CD40L rather than relying solely on “danger signals” [28,31].

This matters more with OVs than with many “cleaner” vaccine platforms because repeat viral dosing and intratumoral infection create changing antigen hierarchies. Each infection pulse supplies abundant viral proteins plus waves of tumor debris; the immune system naturally forms dominance hierarchies and can actively immunodominate (suppress) subdominant specificities depending on route, tissue context, and antigen presentation kinetics. This is well documented in viral systems where infection route and local priming environments sharpen or reshape immunodominance, and where competition and cross-reactivity can maintain some specificities while eroding others [32,33,34,35,36]. In OVs specifically, repeat dosing is common and biologically meaningful. It is often required for optimal immunotherapy-like activity, and successive doses can recondition the tumor microenvironment via innate immune cell interactions, thereby changing which antigens are seen, when, and in what inflammatory context [30,37]. As such, without intentional design, repeat dosing leads to a high risk of eliciting strong but misfocused T-cell responses and robust antiviral immunity, with only transient or subdominant tumor targeting [29,30].

This is precisely where encoding MHC II-restricted neoepitopes or using long-antigen designs that favor class II processing becomes strategically important. Long peptides and long antigens are preferentially taken up and processed by APCs, increasing the probability that the same construct yields coordinated CD4^+^ and CD8^+^ responses and enabling repeated cycles of DC licensing as therapy continues [38]. Clinically, personalized neoantigen vaccine trials consistently show that polyfunctional CD4^+^ responses are frequent and can be durable, supporting trafficking, effector maintenance, and epitope-spreading properties that are needed to “lock in” when the OV is repeatedly perturbing antigen availability and inflammatory tone [3,4,5]. Preclinical neoantigen vaccine work further supports the notion that prioritizing CD4-activating neoantigens can increase overall vaccine efficacy, consistent with the licensing and maintenance model [39]. Finally, OV-focused work underscores the centrality of competent antigen presentation and cross-priming circuits for durable antitumor immunity during virotherapy, reinforcing why “help + presentation” is a core design axis rather than a nice-to-have in OV therapy [3,40,41].

A second rationale is anchored in tumor heterogeneity and immune escape. Clinical and genomic analyses have suggested that the high clonality of neoantigens present across most tumor cells may correlate with improved immunotherapy sensitivity, motivating selection strategies that prioritize clonality and expression rather than merely high predicted binding affinity [42]. For neoantigen-encoded OVs, this prioritization is practical because payload capacity is limited, insert stability can degrade as cassette complexity/size increases under replication-associated selection pressures, and clinical-grade manufacturing constraints favor a focused set of high-value epitopes [27,43,44]. Encoding clonal, expressed neoantigens can therefore deliver a higher “return on payload” by maximizing the fraction of tumor cells carrying the target and reducing opportunities for immune-driven outgrowth of antigen-negative subclones [42,45,46].

## 3. Neoantigen Selection and Prioritization Optimized for OV Payloads

Neoantigen selection for OV encoding differs subtly from selection for peptide or RNA vaccines because viral biology imposes constraints on payload size, genomic stability, transcriptional timing, and the need to preserve viral fitness while offering unique advantages such as localized inflammation, antigen release, and the possibility of repeat intratumoral dosing. A practical pipeline therefore emphasizes not only immunogenicity potential but also “encode-ability”: the likelihood that a chosen antigen format can be stably inserted, expressed at sufficient levels in infected tumor cells, and processed/presented under inflammatory conditions.

Variant discovery typically starts with matched tumor–routine sequencing to identify somatic SNVs and indels, augmented by structural variant and fusion calling when relevant. Because predicted neoantigens can be numerous but only a fraction are naturally presented and immunogenic, expression confirmation is essential. RNA sequencing can confirm transcription of mutant alleles, quantify expression levels, and support prioritization toward targets more likely to be presented. Integrating clonality estimates further enriches for targets less prone to immune escape and may be particularly valuable when payload capacity is limited in specific OV platforms [1,2,42].

HLA typing is a foundational step because peptide presentation is allele-restricted. Both DNA- and RNA-based approaches can be used, and the output feeds into computational prediction of peptide-MHC binding, antigen processing, and presentation likelihood. Importantly, multiple analyses have emphasized persistent limitations in purely in silico pipelines, including false positives in binding predictions, incomplete modeling of processing and transport, and historically weaker class II prediction relative to class I [47,48,49,50,51]. Contemporary tools increasingly integrate mass spectrometry-derived ligand data to improve performance, such as NetMHCpan [41], as well as presentation-focused models like MHCflurry 2.0 that incorporate antigen processing features [52]. Even so, the field broadly converges on a combined computational and experimental validation setting when resources and timelines permit, particularly for high-stakes clinical encoding decisions [41,52].

A recurring lesson from neoantigen vaccine trials is that durable control likely depends on coordinated CD4^+^ helper and CD8^+^ effector responses. In the NeoVax melanoma study, vaccine-induced CD4^+^ responses were frequent and polyfunctional, and CD8^+^ responses were also observed with evidence of mutant specificity and tumor recognition [3]. The first-in-human personalized RNA “mutanome” vaccine similarly demonstrated broad, poly-specific immunity in melanoma [4]. For OV payloads, this motivates deliberate inclusion of both class I- and class II-restricted neoepitopes, or antigen designs (i.e, long peptides and minigenes) that favor class II processing, thereby improving DC licensing and memory formation in the setting of intense innate stimulation [3,4,41].

A key refinement for OV programs is to prioritize (presented, not just predicted) neoantigens using tiered validation. Immunopeptidomics MHC ligand elution followed by mass spectrometry can directly confirm natural presentation, providing a higher-confidence subset when feasible. Where patient material allows, in vitro T cell assays using peripheral blood mononuclear cells can assess immunogenicity and mutant specificity, while tumor organoid or autologous target-killing assays can add functional relevance. Because these assays can be time-intensive, a pragmatic approach is to apply them selectively to the top-ranked candidates that are most likely to be encoded, rather than attempting exhaustive validation across the full candidate list [1,41].

Clinically, individualized nucleic-acid platforms have demonstrated feasibility for multi-epitope encoding. In KEYNOTE-942, individualized mRNA therapy mRNA-4157 (V940) encoded up to dozens of neoantigens and improved recurrence-free outcomes when combined with pembrolizumab in resected high-risk melanoma [53]. For OVs, optimal numbers depend on insert capacity and stability of the specific backbone. A practical approach is to encode a focused set (often ~10–30 epitopes) spanning class I and class II, prioritized by expression and clonality, and to reserve “long-tail” epitopes for heterologous boosts (e.g., mRNA or peptide) if a prime–boost strategy is envisioned [1,53].

## 4. Engineering Strategies: Antigen Formats, Expression Timing, and Platform Choices

Designing neoantigen payloads for OVs requires aligning antigen format with the biology of viral transcription and replication, and host antigen processing. Three payload archetypes are commonly considered. The first is a string of minigenes encoding multiple neoepitopes, often separated by protease cleavage motifs or linker sequences to promote processing. This format is compact and scalable but can be vulnerable to immunodominance, where a subset of epitopes captures the majority of the response, potentially suppressing breadth. The second is long-antigen constructs (long peptides or extended mutant regions) designed to enhance class II presentation and helper priming; these may be larger and require careful stability testing, but can better support durable immunity. The third is hybrid designs that encode both selected class I epitopes and longer class II-favoring segments, attempting to balance compactness and helper breadth [3,41].

Promoter selection and expression timing can materially affect immunogenicity. Early expression may enhance direct presentation by infected tumor cells, whereas late expression can coincide with extensive viral replication and cell lysis, potentially favoring cross-presentation by APCs. Because different OV backbones have distinct transcriptional kinetics and immune antagonism profiles, “one-size-fits-all” rules rarely apply; instead, stability and expression should be empirically evaluated for each platform. Net effect matters clinically: a payload that is theoretically immunogenic but poorly expressed in infected tumors will underperform relative to a smaller, stably expressed cassette that is consistently presented [27].

Platform choice determines not only payload capacity but also route of delivery, antiviral immunity constraints, tumor tropism, and safety considerations. HSV-based OVs offer large genomes with substantial insert capacity and extensive clinical experience, exemplified by T-VEC [8] and G47Δ [12,13]. Vaccinia viruses have high cytoplasmic replication capacity and can accommodate transgenes; JX-594 demonstrated dose-related survival signals and immune activation in HCC [9], and later phase III-scale efforts informed the field’s understanding of patient selection and endpoints [10]. Adenovirus backbones offer manufacturability advantages and can be engineered for tumor selectivity, whereas reovirus (pelareorep) has a distinct biology, with systemic delivery experience and combination trial data across multiple settings [11]. Selection should therefore be indication-driven: injectable melanoma lesions, intracranial disease, liver-dominant tumors, and disseminated metastatic disease each impose different requirements on delivery feasibility and risk tolerance [8,10,11,12,13].

Antiviral immunity is a central engineering constraint. Repeated dosing can be limited by neutralizing antibodies and rapid innate clearance, especially for systemically delivered viruses. Intratumoral delivery can partially circumvent this by achieving high local multiplicity of infection, but even then, antiviral T cells may shorten expression windows. Strategies to mitigate this include transient immunomodulation, use of less prevalent serotypes, capsid and envelope engineering, or heterologous prime–boost designs in which an antigen-agnostic OV primes the tumor microenvironment quickly, followed by a later personalized antigen-bearing agent that boosts mutation-specific immunity. Such sequencing aligns with the reality that customized neoantigen selection and viral manufacturing take time, and it exploits the complementary kinetics of platform readiness and individualized design [1,27].

## 5. Clinical Translation: Workflow, Evidence, and Trial Design

The clinical case for neoantigen-encoded OVs is supported by two mature evidence streams: first, OVs can produce clinical benefit and measurable immune activation; second, personalized neoantigen vaccines can expand mutation-specific T cells in humans. The T-VEC phase III OPTiM trial established durable response benefit in advanced melanoma [8], while G47Δ demonstrated clinically meaningful outcomes in glioblastoma trials and led to conditional approval in Japan [12,13]. Across other backbones, DNX-2401 (adenovirus) in glioma, pexa-vec in hepatocellular carcinoma development, and pelareorep in combination regimens have collectively shaped translational expectations regarding dosing, immune monitoring, and endpoint selection [10,11]. In parallel, melanoma neoantigen vaccines (peptide and RNA) demonstrated feasibility, breadth, and polyfunctional immunity [3,4], and later individualized approaches continued to show immunogenicity and clinical promise, including KEYNOTE-942, where individualized neoantigen therapy improved outcomes when combined with PD-1 blockade [53]. 

**Figure 1:**
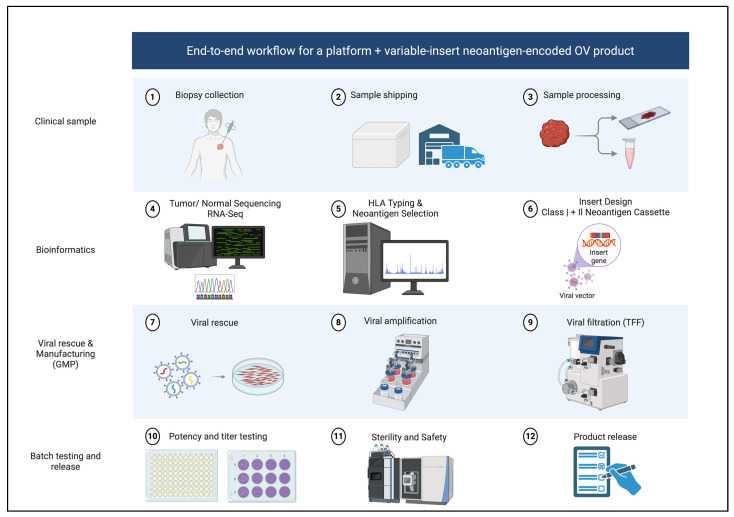
End-to-end workflow for a platform + variable-insert neoantigen-encoded OV product. Clinical sampling includes biopsy collection, shipment, and processing. Bioinformatics comprises tumor/normal sequencing with RNA-seq, HLA typing, and neoantigen prioritization. The variable insert is designed as a class I + class II neoantigen cassette and introduced into a fixed OV backbone, followed by viral rescue, amplification, and downstream processing (e.g., TFF). Batch testing includes potency/titer testing and sterility/safety assays prior to product release. Created in BioRender. Alkayyal, A. (2026) https://BioRender.com/ilqtl2l (accessed on 20 February 2026).

A practical workflow for neoantigen-encoded OV therapy begins with patient selection guided by delivery feasibility and immunologic need. For intratumoral programs, accessible injectable lesions are essential. In contrast, systemic delivery requires evidence that the chosen backbone can reach and infect relevant tumor sites with acceptable safety. Tumor types with low baseline T cell infiltration and poor checkpoint responsiveness are attractive settings if the OV backbone has an appropriate risk profile. Tumor sampling then typically requires fresh biopsy or surgical material suitable for whole-exome or whole-genome sequencing plus RNA sequencing, alongside matched germline. Baseline immune profiling, including CD8 density, interferon signatures, myeloid programs, and antigen presentation markers, can support stratification and interpretation of response heterogeneity. Neoantigen selection follows the clonality, expression, and prediction logic described above, with class II inclusion where feasible. Manufacturing then proceeds through insert design, cloning, viral rescue, stability and potency testing, and GMP release assays, including identity, infectious titer, sterility, endotoxin, replication competence as relevant, and insert integrity by sequencing. Finally, treatment schedules and monitoring plans are implemented with an appreciation of antiviral immunity; intratumoral regimens often use early, weekly or biweekly dosing, with combination checkpoint blockade considered when mechanistically justified [1,27]. This end-to-end clinical and GMP workflow is summarized in Figure 1.

Combination therapy is plausible but not automatically beneficial. The experience with T-VEC plus pembrolizumab illustrates this complexity. However, although earlier phase studies suggested promising activity, a significant randomized phase III study did not demonstrate improvement in progression-free or overall survival over pembrolizumab alone [54]. This outcome emphasizes that synergy depends on the context of the disease stage, lesion injectability, baseline immunity, timing, and potentially the choice of payload, and it argues for biomarker-guided enrollment rather than assuming universal benefit from OV–checkpoint pairing. Neoantigen encoding may refine this equation by increasing the proportion of mutation-focused responses and enhancing the probability of epitope spreading. Still, these hypotheses require prospective validation in rationally designed trials [1,54].

Immune monitoring endpoints should be chosen to distinguish mere inflammation from productive, mutation-specific immunity. In blood, neoantigen-specific T cells can be tracked by ELISpot, activation-induced marker assays, intracellular cytokine staining, and multimer staining when feasible. TCR sequencing can quantify clonal expansion and provide evidence of tumor trafficking when paired with tumor biopsies. In the tumor, paired pre- and post-biopsies can assess spatial CD8 infiltration, DC activation signatures, myeloid polarization, interferon programs, and antigen-presenting machinery. A particularly valuable endpoint is evidence of epitope-spreading responses to non-encoded neoantigens, which may correlate with systemic control and reduced escape. Clinical neoantigen vaccine data demonstrate that epitope spreading can occur in humans, including in NEO-PV-01 programs where spread to non-vaccinated neoantigens was observed [3,7]. Finally, response criteria should accommodate immune-related patterns; pseudoprogression and delayed responses have been described in immunovirotherapy contexts and can confound early radiographic readouts if not anticipated [55].

## 6. Regulatory and GMP Considerations for “Platform Plus Variable Insert” Products

Personalized neoantigen-encoded oncolytic viruses (OVs) sit at the intersection of virotherapy, gene therapy, like genetic engineering, and individualized cancer vaccination. A central regulatory concept for feasibility is the “platform plus variable insert” paradigm, in which the viral backbone, manufacturing process, and release assays are standardized. At the same time, the patient-specific neoantigen cassette is the only variable element. This logic aligns with how regulators and developers have begun to think about individualized cancer vaccines more broadly—where the manufacturing workflow is fixed, but the encoded sequences differ from patient to patient—provided that critical safety and quality attributes and clinical risk remain comparable across lots [1,53,56]. In practice, this pushes development toward a master platform strategy: lock down the backbone, insertion locus, promoter designs, and upstream/downstream processes early, then treat each personalized insert as a controlled variant within a validated performance envelope [27,44].

From a GMP perspective, the platform claim must be earned by demonstrating process consistency, product identity, and functional comparability across representative inserts. Reviews on clinical translation of OVs emphasize that manufacturing is not merely scaled laboratory work; replication-competent viral products require rigorous control of raw materials, cell substrates, adventitious agent testing, and contamination risk, alongside careful viral genetics oversight because the product can evolve under selective pressure [27,57]. Broader experience in viral vector manufacturing also underscores capacity constraints, lot-to-lot variability risks, and the need for robust analytics and standardized unit operations to ensure reproducibility, which becomes even more acute when the product is individualized [58,59].

Insert stability becomes a defining GMP risk for neoantigen-encoded OVs, because inserted sequences can be lost, rearranged, or selected against during amplification and passaging, especially when inserts are long or burdensome to viral fitness. A practical GMP development program therefore needs explicit genetic stability packages: serial passaging under manufacturing-like conditions with sequence confirmation of the insert and junctions, and predefined acceptance criteria for integrity at release and over shelf life [27,44]. For individualized products, comparability also depends on constraining the design space: limiting insert length, using standardized linkers and processing motifs, and minimizing patient-to-patient variability in cassette structure even if the epitope sequences differ [1,56].

Potency is another area where infectious titer alone is rarely sufficient for a credible GMP control strategy in neoantigen-encoded OVs. Because the intended mechanism includes both direct oncolysis and immunologic programming, potency frameworks increasingly advocate a matrix approach: infectivity, a qualified readout of transgene (neoantigen) expression, and, where feasible, a cell-based functional surrogate that captures biological activity relevant to the mechanism [27,60]. While fully personalized immune-potency assays are often unrealistic at release, qualified platform assays that measure antigen cassette expression kinetics or antigen processing can strengthen the argument that each individualized lot is not only infectious but also immunologically competent as designed [60].

Finally, GMP planning must incorporate biosafety-linked clinical requirements, especially biodistribution and shedding considerations that vary by backbone and route (intratumoral versus systemic). Many clinical translation studies of oncolytic virotherapy highlight that replication-competent vectors often require predefined monitoring plans for shedding and transmission risk, along with route-specific risk mitigation and patient precautions, all of which must be supported by platform data generated early enough to inform trial conduct and labeling [27,57]. Cancer vaccine-specific regulatory discussions also emphasize that clinical development packages are judged not only by immunogenicity but also by product characterization, control strategy, and risk-based safety planning elements, which become more complex when the product is individualized [56,61].

## 7. Challenges and Future Directions

A persistent barrier to personalized neoantigen immunotherapies is the timeline: sequencing, bioinformatics, insert design, cloning/rescue, release testing, and scheduling must converge quickly enough that patients can receive the product while disease kinetics still allow benefit. Clinical experience with individualized neoantigen vaccines shows that feasibility improves when workflows are industrialized with standardized wet-lab steps, parallelized compute pipelines, and predefined QC gates rather than modified decision-making on a per-patient basis [1,56]. Importantly, clinical proof that viral-vector and nucleic-acid personalized vaccines can be operationalized at meaningful speed is accumulating, supporting the idea that neoantigen-encoded OVs could be practical if the platform is engineered for rapid insertion and the manufacturing train is built for short cycle times [62,63]. A forward-looking strategy is to formalize “bridging regimens,” where a rapid, antigen-agnostic OV or a fixed immunostimulatory OV initiates local inflammation and priming, followed by the personalized neoantigen-encoded OV (or heterologous boost) when ready, essentially decoupling “start immunotherapy now” from “deliver personalization later,” while still aiming for immunologic coherence [1,56].

## 8. Conclusions

Neoantigen-encoded OVs represent a logically convergent strategy that combines tumor-localized inflammatory “in situ vaccination” with deliberate, patient-specific antigen programming. The clinical maturation of oncolytic virotherapy including phase III evidence for T-VEC in melanoma and clinical efficacy signals for other backbones such as G47Δ (teserpaturev) in glioblastoma supports the feasibility of deploying replication-competent viruses as immunotherapy agents, not merely as cytolytic drugs [8,64]. In parallel, contemporary personalized neoantigen vaccines have demonstrated that individualized mutation-derived targeting can reproducibly expand neoantigen-specific T cells in patients and can deliver clinical benefit in at least some settings, reinforcing the central premise that “private” tumor mutations are actionable immune targets [7,53,65].

The next wave of progress for neoantigen-encoded OVs will likely depend on four practical determinants. First, improved neoantigen selection that explicitly accounts for clonality and heterogeneity because clonal neoantigens are more likely to provide durable targets, while heterogeneity and immunodominance can undermine otherwise elegant designs [42,46]. Second, platform engineering that preserves insert stability and expression without compromising viral fitness, supported by stability-aware cassette design and validated insertion architectures [27,44]. Third, biomarker-driven combination regimens that acknowledge that checkpoint blockade synergy is context-dependent, as illustrated by the negative phase III experience with T-VEC plus pembrolizumab, while still leveraging strong mechanistic rationales for selected combinations such as PD-(L)1 blockade, myeloid modulation, or radiotherapy where indicated [54,66,67,68]. Fourth, streamlined GMP manufacturing pathways that can reliably compress the biopsy-to-dose interval, building on emerging demonstrations that individualized viral-vector and nucleic-acid vaccine workflows can be operationalized within clinically meaningful timelines [56,62,63].

If these constraints are addressed, neoantigen-encoded OVs could mature into truly personalized “living vaccines,” capable of turning local viral infection into systemic, durable tumor control through a combination of programmed neoantigen priming and OV-driven epitope spreading [69,70].

## Data Availability

No new data were created or analyzed in this study. Data sharing is not applicable to this article.
